# Novel compound C150 inhibits pancreatic cancer through induction of ER stress and proteosome assembly

**DOI:** 10.3389/fonc.2022.870473

**Published:** 2022-10-05

**Authors:** Tao Wang, Ping Chen, Scott Weir, Michael Baltezor, Frank J. Schoenen, Qi Chen

**Affiliations:** ^1^ Department of Pharmacology, Toxicology and Therapeutics, University of Kansas Medical Center, Kansas, KS, United States; ^2^ Department of Cancer Biology, University of Kansas Medical Center, Kansas, KS, United States; ^3^ Biotechnology Innovation and Optimization Center, University of Kansas, Lawrence, KS, United States; ^4^ Higuchi Biosciences Center, University of Kansas, Lawrence, KS, United States; ^5^ Medicinal Chemistry Core Laboratory, Lead Development and Optimization Shared Resource, University of Kansas Cancer Center, Lawrence, KS, United States

**Keywords:** pancreatic cancer, ER stress, cell senescence, proteasome, autophagy

## Abstract

Pancreatic cancer is a devastating disease with a dismal prognosis and poor treatment outcomes. Searching for new agents for pancreatic cancer treatment is of great significance. We previously identified a novel activity of compound C150 to inhibit pancreatic cancer epithelial-to-mesenchymal transition (EMT). Here, we further revealed its mechanism of action. C150 induced ER stress in pancreatic cancer cells and subsequently increased proteasome activity by enhancing proteasome assembly, which subsequently enhanced the degradation of critical EMT transcription factors (EMT-TFs). In addition, as cellular responses to ER stress, autophagy was elevated, and general protein synthesis was inhibited in pancreatic cancer cells. Besides EMT inhibition, the C150-induced ER stress resulted in G2/M cell cycle arrest, which halted cell proliferation and led to cellular senescence. In an orthotopic syngeneic mouse model, an oral dose of C150 at 150 mg/kg 3× weekly significantly increased survival of mice bearing pancreatic tumors, and reduced tumor growth and ascites occurrence. These results suggested that compound C150 holds promises in comprehensively inhibiting pancreatic cancer progression.

## Introduction

Pancreatic cancer is among the most malignant types of cancers and will soon become the third leading cause of cancer-related death in the United States (1). The current estimated overall 5-year survival rate is only 10% (1). Despite our increasing understanding of the genetic and molecular makeups of the disease over the past few decades, the prognosis of pancreatic cancer remains very poor. Current first-line chemotherapy options include gemcitabine plus nab-paclitaxel and the regimen of FOLFIRINOX (oxaliplatin, irinotecan, fluorouracil, and leucovorin). These therapies improved the median survival by a few months compared to gemcitabine mono treatment, but they added significant toxicities to patients (2, 3).

The homeostasis between protein loading and protein folding in the endoplasmic reticulum (ER) is essential for cell survival. Cellular insults that perturb the homeostasis lead to misfolded protein accumulation and ER stress (4). In response to ER stress, cells activate the unfolded protein response (UPR) pathways to restore homeostasis in the ER, as a survival mechanism (5). The UPR is controlled by three ER membrane-bound proteins, protein kinase RNA-like ER kinase (PERK), inositol-requiring protein 1α (IRE1α), and activating transcription factor 6 (ATF-6) (5). Activation of PERK, IRE1α, and ATF-6 activates their direct downstream transcription factors, ATF-4, XBP1-s, and spliced-ATF-6, respectively, leading to increased gene expressions of chaperone proteins to enhance protein folding capacity in the ER (6, 7). In addition, PERK activation results in the attenuation of mRNA translation through eIF2α phosphorylation, therefore reducing new protein load to the ER (8). Finally, ER stress also activates the ER-associated degradation (ERAD) pathway to facilitate misfolded protein removal through the ubiquitin–proteasome system and autophagy (9, 10). By increasing the level of protein folding chaperones, reducing protein synthesis, and enhancing protein removal through ERAD, the UPR signaling functions as a pro-survival mechanism to restore ER homeostasis (10). However, severe or prolonged ER stress that goes beyond the UPR rescue capacity would lead to cell proliferation arrest, cell death, and/or senescence (11–13).

As the major protein degradation system in the cell, proteasome levels and activities are often elevated upon ER stress, to facilitate the clearance of misfolded or damaged proteins (14). The two major forms of proteasomes in the mammalian cells are 20s proteasome and 26s proteasome, both of which are multi-subunit protein complexes. The 20s proteasome is made up of two sets of α subunits (α_1-7_) and two sets of β subunits (β_1-7_) with a stoichiometry of α_1-7_β_1-7_β_1-7_α_1-7_, while the 26s proteasome is composed of a 20s proteasome flanked at one or both ends by a 19s regulatory particle (19s RP) that is made up of 19 different subunits (15). Therefore, the assembly of a full 26s proteasome requires the steps of 20s proteasome assembly, 19s RP assembly, and the docking of 19sRP to the 20s proteasome (16). The 26s proteasomes serve as the main complex for cellular protein degradation in an ATP- and ubiquitin-dependent manner (17), while the 20s are also capable of degrading a portion of cellular proteins independent of ATP and ubiquitin (18, 19).

Because of the essential role of ER balance in cell survival, disrupting ER balance has been proposed as a potential therapeutic approach in cancer treatment (20, 21). We have previously reported that a quinoline compound (namely, C150) enhanced the proteasome-mediated degradation of Snail protein in pancreatic cancer cells, causing EMT inhibition and reduced cancer cell invasion (22). In this study, we further revealed that C150 induced profound ER stress in pancreatic cancer cells and led to the increase of proteasome assembly, cellular autophagy, and attenuation of general mRNA translation. C150 treatment arrested pancreatic cancer cells in the G2/M phase, induced cellular senescence, and increased cellular sensitivity to gemcitabine treatment. C150 treatment significantly increased survival and reduced tumor growth in a syngeneic pancreatic cancer mouse model.

## Materials and methods

### Cell culture and reagents

Human pancreatic cancer cells PANC-1 and MIA PaCa2 were obtained from the American Type Culture Collection (ATCC). Murine pancreatic cancer Pan02 cells were generously donated by Dr. Shrikant Anant from the University of Kansas Medical Center. Cells were cultured in DMEM (10-013-CV, Corning Life Sciences) with 10% FBS (F0926, Sigma-Aldrich) and 100 units/ml penicillin/streptomycin (30-001-CI, Corning Life Sciences) in a 37°C cell incubator with humidified 5% CO_2_. All cells were cultured within 20 passages in our laboratory. Compound C150 was purchased from ChemBridge Chemical Library (ChemBridge, San Diego, CA) and stocked in dimethyl sulfoxide (DMSO). All C150 treatments were diluted in cell culture medium with a final DMSO concentration lower than 0.1% (v/v%). Control cells were treated with the same concentrations of DMSO with respect to drug-treated groups (<0.1% v/v%).

### Proteasome activity assay

PANC-1 cells were seeded and grown in 100-mm petri dishes at 1 × 10^6^ cells/dish. The next day, medium was replaced with fresh medium containing C150 or DMSO, and cells were treated for 24 h. After treatment, cells were lysed in proteasome activity lysis buffer (50 mM HEPES, 10 mM NaCl_2_, 1.5 mM MgCl_2_, 1 mM EDTA, 2 mM ATP, and 1% Triton X-100) on ice for 1 h. The supernatants of the cell lysates were collected by centrifugation at 16,000 × *g* for 15 min and kept on ice. The Pierce BCA protein assay (23225, Thermo Scientific) was performed to determine protein concentrations in the cell lysates. In a black-wall 96-well plate, 150 µl of fluorescent proteasome substrate solution Suc-LLVY-AMC (BML-P802-0005, Enzo Life Science, Farmingdale, NY) and 50 μl of cell lysates were added per well to a final substrate concentration of 100 μM. The proteasome inhibitor epoxomicin (4 μM) was added in the negative control group. The plate was then placed in a fluorescent plate reader with 37°C incubation and read under the kinetic mode at 360/460 nm every 20 min for 80 min. Fluorescent readings were then normalized to the protein amounts in the cell lysates of each sample.

### Western blots

Cells were lysed in Pierce RIPA buffer (89901, Thermo Scientific) supplemented with protease and phosphatase inhibitor cocktails (P8340, P5726, and P0044, Sigma-Aldrich). Protein concentrations of the lysates were determined using the Pierce BCA protein assay (23225, Thermo Scientific). The cell lysates were then mixed with 2× Laemmli SDS loading buffer (161-0737, Bio-Rad), run in 8% or 10% SDS-PAGE gel, and transferred onto 0.2-μm PVDF membranes (ISEQ00010, MilliporeSigma). Membranes were blocked in 5% blocking grade milk in 0.1% TBST solution (0.1% Tween-20 in 1× TBS) for 2 h at room temperature, then incubated with primary antibody at 4°C overnight in 5% BSA/0.1% TBST solution. Mouse monoclonal antibodies against 20s subunits β-1 (sc-374405), β-2 (sc-58410), β-5 (sc-393931), α-5 (sc-137240), α-6 (sc-271187), and 19s subunits PSMC-2 (sc-166972), PSMC-3 (sc-100462), and PSMC-4 (sc-166115) were purchased from Santa Cruz Biotechnology (Dallas, TX). Rabbit monoclonal antibodies against Bip (3177T), ATF-4 (11815S), ATF-6 (65880T), XBP-1s (40435S), LC-3 (3868S), phospho-eIF2α (3398T), Vinculin (4650S), and GAPDH (2118S) were purchased from Cell Signaling Technology (Danvers, MA). Mouse monoclonal anti-puromycin (PMY-2A4) and anti-eIF2α (PCRP-EIF2S1-1E2) antibodies were purchased from Developmental Studies Hybridoma Banks (the University of Iowa, Iowa City, IA). Following primary antibody incubation, membranes were washed three times in 0.1% TBST solution and then incubated with HRP-linked anti-rabbit (7074S) or anti-mouse (7076S) secondary antibodies (Cell Signaling Technology, Danvers, MA) in 5% milk for 2 h at room temperature. Blotting bands were then detected by using Pierce ECL plus reagents (32132, Thermo Scientific).

### Native gel analysis for assembled proteasome

PANC-1 cells were seeded and treated the same way as in the proteasome activity assay. After treatments, cells were lysed in proteasome activity assay lysis buffer on ice for 1 h. The supernatants of the cell lysates were collected by centrifugation at 16,000 × *g* for 15 min and kept on ice. After determining the protein concentrations in the lysates with Pierce BCA protein assay, the samples were then mixed with 2× non-denaturing loading buffer (161-0738, Bio-Rad). A total of 30 μg of protein from each sample was loaded and separated in 4% Tris-Borate native gel at 100 V for 3.5 h in running buffer (89 mM Tris, 89 mM boric acid, 2 mM EDTA, 5 mM MgCl_2_, and 1 mM ATP). The 4% Tris-Borate native gels were made as follows (10 ml): 7.5 ml of H_2_O + 1.333 ml of 30% polyacrylamide (1610158, Bio-Rad) + 50 μl of MgCl_2_ (1 M) + 100 μl of ATP (0.1 M) + 1 ml of 10× Tris-Boric-EDTA buffer (161-0733, Bio-Rad) + 100 μl of 10% APS + 10 μl TEMED. After electrophoresis, gels were soaked in 1× Tris-Glycine buffer with 0.1% SDS for 30 min. Proteins in gels were then transferred onto 0.2 μm PVDF membranes at 100 V for 3.5 h at 4°C in transferring buffer (1× Tris-Glycine buffer with 20% methanol). Subsequently, membranes were stained with Ponceau S to reveal protein bands, then washed in 0.1% TBST solution and blocked in 5% blocking grade milk for 2 h at room temperature before incubating with anti-20s β-5 antibody and anti-19s PSMC-3 antibody for overnight at 4°C. The membrane was then washed in 0.1% TBST buffer and incubated with HDR-conjugated anti-mouse secondary antibody (7076S, Cell Signaling Technology, Danvers, MA). Protein bands were detected with Pierce ECL plus reagents (32132, Thermo Scientific).

### RT-qPCR

Total RNA was extracted from cells using TRIZOL reagents (AM9738, Invitrogen) according to the manufacturer’s protocol. The synthesis of cDNA was carried out with 1 μg of total RNA using the OneScript cDNA Synthesis Kit (G234, Applied Biological Materials, Richmond, BC, Canada). cDNA was then diluted five times in nuclease-free H_2_O for RT-qPCR reaction. RT-qPCR was performed using the Bio-Rad iQ iCycler detection system with One-Step BrightGreen reagents (MasterMix-S, Applied Biological Materials, Richmond, BC, Canada) according to the manufacturer’s protocol. Each reaction was carried out in 10 μl volume with 5 μl of 2× BrightGreen qPCR MasterMix, 0.6 μl of forward and reverse primer mix (10 μM), 2 μl of diluted cDNA, and 2.4 μl of nuclease-free H_2_O. All qPCR reactions were run under the following cycling conditions according to the protocol from the kit: enzyme activation at 95°C for 10 min, followed by 40 cycles of denaturation (95°C for 15 s) and annealing/extension (60°C for 60 s). The melting curve was detected at 55°C–95°C with 0.5°C increments. Three independent experiments were carried out, and reactions were run in triplicate for each sample. Gene expression was quantified using the 2^ΔΔ-Ct^ method with GAPDH as the internal control gene. Primers for detected genes are listed in **Table 1**.

**Table 1:** Gene primer sequences for RT-qPCR.

Gene name	Forward (5’ -> 3’)	Reverse (5’ -> 3’)
**PSMG-1 (PAC-1)**	TCC TTT CCT GAG AGC CCT AAA A	TGT TCT AGC AAT GGA CAA CAC G
**PSMG-2 (PAC-2)**	ACC GAT TGT CTT GTG CCA ATG	AGG CAA TGA ATA CAC TTC AGC AT
**PSMG-3 (PAC-3)**	GAA GAC ACG CCG TTG GTG ATA	GAA GGA CTT TTG TGG TGA GCA
**PSMG-4 (PAC-4)**	GTC CAC TTC CAC GTC ATG C	GGG AGG TAG ACA CGG GGA T
**POMP**	ACT TGG ATC TGA GCT AAA GGA CA	GGG GAT GAC TAG GCA AAA GTT C
**PAAF-1**	GGA GGT CTT GGT GTG TCT TCT	CAA CGA TGG CTG TAT CCA GGA
**PSMD-10**	GGG TGT GTG TCT AAC CTA ATG G	GGC CAG AAT ACT CTC CTT CAA CT
**PSMD-5**	GCG CTG CTG AGA GAG GTA G	AGT CTT TTC CCT ATG GTT CTC GT
**PSMD-9**	AGG AGG AGA TAG AAG CGC AGA	GTG CGG ACT TGG TAC AGG T
**IL6**	CCCCTCAGCAATGTTGTTTGT	CTCCGGGACTGCTAACTGG
**IL7**	CCCTCGTGGAGGTAAAGTGC	CCTTCCCGATAGACGACACTC
**IL-13**	CCTCATGGCGCTTTTGTTGAC	TCTGGTTCTGGGTGATGTTGA
**IL-15**	TTTCAGTGCAGGGCTTCCTAA	GGGTGAACATCACTTTCCGTAT
**CCL5**	CCAGCAGTCGTCTTTGTCAC	CTCTGGGTTGGCACACACTT
**CXCL10**	GTGGCATTCAAGGAGTACCTC	TGATGGCCTTCGATTCTGGATT
**PAI-1**	CCACCTCCGTGAAGGAATGAC	GGTAGTGTGGCATAAACAGCA
**TNF-a**	CCTCTCTCTAATCAGCCCTCTG	GAGGACCTGGGAGTAGATGAG
**MCP1**	CAGCCAGATGCAATCAATGCC	TGGAATCCTGAACCCACTTCT
**GAPDH**	CCA GGT GGT CTC CTC TGA CTT CAA CA	AGG GTC TCT CTC TTC CTC TTG TGC TC

### Cell cycle analysis

PANC-1 cells were seeded and grown in 60-mm petri dishes at 5 × 10^5^ cells/dish. The next day, the medium was changed into fresh medium with C150 or DMSO. At 24 h or 48 h, cells were collected by trypsinization, washed with 1× PBS twice, and fixed in 70% ethanol at −20°C overnight. Cells were then washed with 1× PBS and stained in PI staining solution (20 μg/ml propidium iodide in 1× PBS solution with 0.1 mg/ml RNase A and 0.1% Triton X-100) at 37°C for 15 min protected from light. Cells were kept in the staining solution overnight at 4°C protected from light before being analyzed for cell cycle distribution with flow cytometry (BD LSR II, BD Biosciences).

### Cell growth curve by MTT assay

Cells were seeded in 96-well plates at 5,000 cells/well and incubated in the cell culture incubator overnight. The next day, the medium was changed into fresh medium containing C150 at the indicated concentrations. Cells were further incubated for 0, 24, 48, 72, and 96 h, then MTT (3-(4,5-dimethylthiazol-2-yl)-2,5-diphenyltetrazolium bromide) was added to each well to a final concentration of 0.5 mg/ml, and the plates were further incubated for 4 h. The medium was then removed and 150 μl of DMSO was added to each well. Absorbance was measured at 570 nm using a microplate reader (BioTek, Winooski, Vermont).

### Gemcitabine combination treatment

PANC-1 cells were seeded in 96-well plates at 5,000 cells/well and incubated overnight, and then changed into fresh medium with treating drug combinations in a matrix design as shown in **Figure 3D**. Cells were treated for 72 h and viability was detected by MTT assay. The combination index was calculated according to Chou-Talalay’s method using CompuSyn software (23).

### Immunofluorescent staining for LC-3 puncta

PANC-1 cells were seeded and grown in eight-chamber microscope cell culture slides (PEZGS0816, MilliporeSigma) at 6 × 10^4^ cells per chamber. The next day, the medium was replaced with fresh medium containing C150 or DMSO, and the cells were treated for 24 h. After treatment, cells were washed twice with 1× PBS and fixed in ice-cold 100% methanol at −20°C for 15 min. Cells were then washed three times with 1× PBS and blocked in 5% normal goat serum (5425S, Cell Signaling Technology, Danvers, MA) in 1× PBS with 0.3% Triton X-100 for 1 h at room temperature. Cells were then incubated with anti-LC-3 antibody (3868S, Cell Signaling Technology, Danvers, MA) at 4°C overnight in antibody incubation buffer (1× PBS with 0.3% Triton X-100 and 1% BSA). After three washes with 1× PBS, cells were incubated with Alexa-488 conjugated secondary antibody (4412S, Cell Signaling Technology, Danvers, MA) for 2 h at room temperature and protected from light. After three washes with 1× PBS, the slide was then coverslipped with the anti-fade mounting solution with DAPI (8961S, Cell Signaling Technology, Danvers, MA) and cured in the dark overnight at room temperature to stain the nuclei before being imaged with fluorescence microscopy at 600× magnification.

### β-galactosidase staining for cellular senescence

The senescence β-galactosidase staining kit (9860S, Cell Signaling Technology, Danvers, MA) was utilized according to the manufacturer’s protocol. Briefly, PANC-1 cells were seeded and grown in 24-well plates at 5 × 10^4^ cells per well. The next day, the old medium in the plates was replaced with fresh medium containing C150 or DMSO, and cells were treated for 24 and 48 h. After treatment, cells in the plates were washed with 1× PBS twice and fixed in 0.5 ml fixative solution for 15 min at room temperature. Following cell fixation, 0.5 ml staining solution with X-gal (1 mg/ml) at pH 6.0 was added to each well. The plates were then sealed with parafilm, wrapped in aluminum foil, and incubated in a 37°C dry oven for 24 h. After removal of the staining solution, cells were washed three times with 1× PBS and covered in 0.5 ml 70% glycerol. At least five random fields per well were imaged using a light microscope under the bright field at 200× magnification. Positively stained cells and the total cells in each image were counted using the multi-point manual counting tool in ImageJ software.

### Syngeneic mouse model of pancreatic cancer

All animal experiments followed an Animal Care and Use Protocol (2018-2443) approved by the Institutional Animal Care and Use Committee at the University of Kansas Medical Center. Female C57BL/6 mice (6–8 weeks old) were purchased from the Jackson Laboratory (Bar Harbor, ME). For tumor cell implantation, mice were put under anesthesia by isoflurane inhalation (5% isoflurane for induction of anesthesia and 2% for maintenance). A subcostal laparotomy was performed to expose the pancreas. A total of 4 × 10^5^ Pan02 mouse pancreatic cancer cells suspended in 50 μl of 1× PBS were injected into the tail of the pancreas. The wound was then sealed with wound clips. Twenty-one days after tumor cell inoculation, two random mice were sacrificed to confirm tumor formation. Subsequently, mice were randomly grouped into two groups (vehicle: *n* = 9, treatment: *n* = 8). Treatments were then commenced with 150 mg/kg of C150 or vehicle (5% Tween-80 + 95% H_2_O) by oral gavage. Mice were treated three times a week for 2 weeks and monitored twice daily for signs of moribund state. The moribund state was determined using body score (<2), or any signs of extreme lethargy, lack of responsiveness to manual stimulus, immobility, or hypothermia. When these signs were observed, the mice were euthanized and counted as death events. Necropsy was then immediately performed, and tumors were weighed and collected. If ascites were present, ascites volume was measured. All survived mice at the endpoint (35 days after tumor inoculation) were euthanized and tumors and ascites were collected upon necropsy.

### Statistics

All data were presented as mean ± SD unless otherwise stated. The Student’s *t*-test was performed for two-group comparisons. One-way ANOVA with the Tukey *post-hoc* test was performed for multi-group comparisons. A *p-*value < 0.05 is considered statistically significant.

## Results

### C150 increased proteasome activity in PANC-1 cells by increasing proteasome assembly

**Figure 1:**
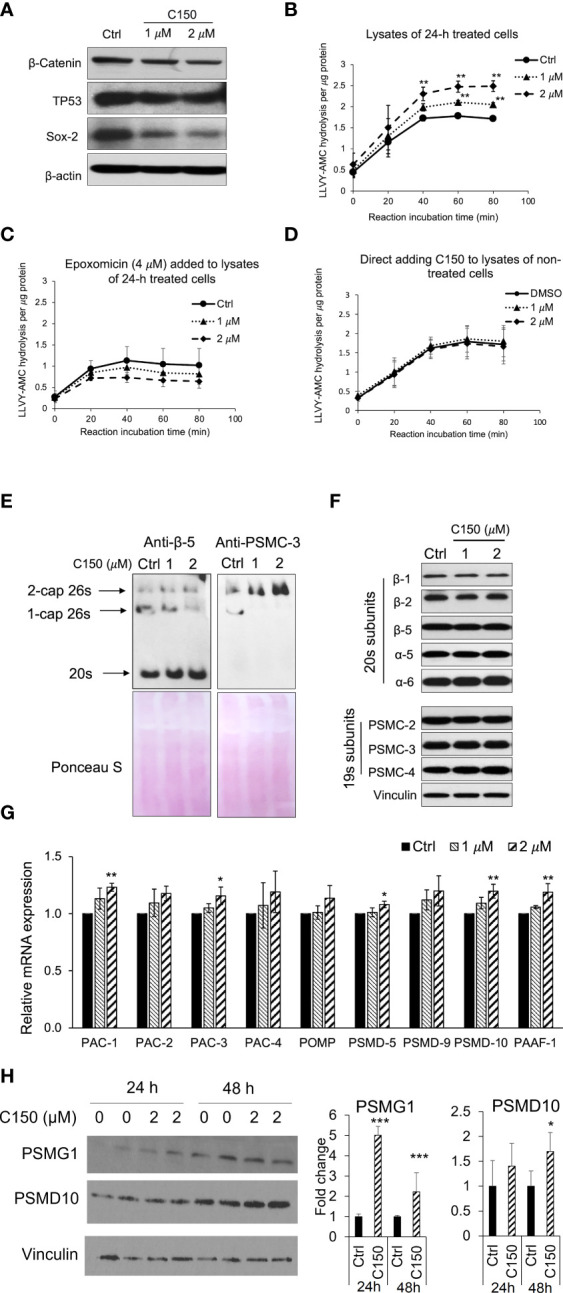
C150 enhanced proteasome activity by increasing 20s proteasome and 26s proteasome assembly. **(A)** C150 decreased β-catenin, TP53, and Sox-2 protein level. PANC-1 cells were treated with DMSO (Ctrl) or C150 (1 μM and 2 μM) for 24 h. Total cell lysate was analyzed. β-Actin was blotted as loading control. **(B, C)** Proteasome activity in PANC-1 cells treated with C150. PANC-1 cells were treated with C150 (1 μM and 2 μM) or DMSO (Ctrl) for 24 h. Cell lysates were collected and incubated with proteasome substrate Suc-LLVY-AMC at 37°C, in the absence **(B)** and presence **(C)** of epoxomicin (4 μM), and the kinetics of fluorescence signal was detected every 20 min for 80 min at 360/460 nm. Fluorescence signal intensity was quantified to the protein amounts in each reaction. **(D)** Proteasome activity in PANC-1 cell lysate incubated with C150. DMSO (Ctrl) or C150 (1 μM and 2 μM) was directly added into lysates of non-treated PANC-1 cells and incubated at room temperature for 30 min before mixing with Suc-LLVY-AMC substrate. **(E)** Native gel blots for assembled 20s and 26s proteasome. Anti b-5 antibody was used to show the 20s and anti-PSMC-3 was used to show the 26s proteasomes. Lower panels show Ponceau S staining. **(F)** Western blots of proteasome subunits. PANC-1 cells were treated with C150 (1 μM and 2 μM) or DMSO (Ctrl) for 24 h. Total cell lysates were used. Vinculin was a loading control. **(G)** RT-qPCR for mRNA expressions of proteasome assembly chaperones. PANC-1 cells were treated with C150 (1 μM and 2 μM) or DMSO (Ctrl) for 24 h. Results were quantified and normalized to Ctrl group using the 2^−ΔΔCt^ method with GAPDH as a housekeeping gene. **(H)** Western blots of PSMG-1 (*PAC-1* gene product) and PSMD-10 proteins. The left panel is a representative image of the Western blots. The right panel shows fold changes of relative bands intensity normalized to Vinculin. All data are presented as mean ± SD of three independent experiments. *p < 0.05, **p < 0.01 , ***p < 0.001 (vs. Ctrl) by either Student’s *t*-tests between two groups, or one-way ANOVA with Tukey HSD tests among multiple groups.

We have previously reported that C150 enhanced the proteasomal degradation of the pro-EMT transcription factor Snail in PANC-1 cells (22). In addition, we found that β-catenin, TP53, and Sox2 protein levels were also reduced by C150 treatment (**Figure 1A**). All of these transcription factors are proteasome substrates (24–26). Therefore, we postulated that C150 increased proteasome activity in the cell. To examine the cellular proteasome activity upon C150 treatment, PANC-1 cells were first treated with C150 (1 μM and 2 μM) for 24 h. The cell lysates were collected under non-denaturing conditions and incubated with a proteasome substrate, Suc-LLVY-AMC, which generates fluorescence upon proteasomal degradation. The results showed that lysates from C150-treated cells exhibited a significantly higher proteasome activity compared to the DMSO-treated group (Ctrl) in a concentration-dependent manner to C150 (**Figure 1B**). This increase was completely attenuated by a specific proteasome inhibitor epoxomicin (**Figure 1C**). To examine whether C150 directly increased the activity of proteasome, non-treated PANC-1 cell lysates were incubated with C150 and the proteasome substrate. The direct incubation of C150 in non-treated cell lysates did not affect proteasome activity (**Figure 1D**). These data suggested that C150-mediated increase in proteasome activity was dependent on a cellular process that requires the integrity of the cell but was not through direct interaction with proteasomes.

We then investigated the total levels of the assembled 20s and 26s proteasome in the PANC-1 cells treated with C150. Anti-β-5 subunit antibody was used to show the 20s proteasomes. Because the 26s proteasome is composed of a 20s proteasome flanked by one or two 19s caps at its ends, an anti-PSMC-3 subunit for 19s RP was also used to show the 1-cap or 2-cap 26s proteasomes. Native gel protein electrophoresis and Western blots showed that the assembled 20s proteasomes and 2-cap 26s proteasomes were both elevated upon C150 treatment (**Figure 1E**). To determine if the increased 20s and 26s proteasome levels were the results of the increased expressions of their subunits, we detected a panel of 20s and 19s subunits by Western blot. All the examined subunits remained unchanged by C150 treatment (**Figure 1F**). Because the abundance of proteasomes in the cell is also regulated by their chaperone-dependent assembly (14, 27), we then examined the expressions of nine proteasome assembly chaperones by RT-qPCR. We found that 24-h C150 treatment (1 μM and 2 μM) significantly increased the expressions of the chaperones PAC-1, PAC-3, PSMD-5, PSMD-10, and PAAF-1, with the other four chaperones showing a trend of increase (**Figure 1G**). We then detected the protein expression of PSMG-1 (PAC-1 gene product) and PSMD-10 as representatives of the increased chaperones. The protein levels of PSMG-1 were increased upon 2 μM C150 treatment at 24 and 48 h (**Figure 1H**), and the protein levels of PSMD-10 were significantly increased at 48 h of treatment (**Figure 1H**). Taken together, the data suggested that C150 enhanced proteasome activity in PANC-1 cells by increasing proteasome assembly.

### C150 induced ER stress, increased autophagy, and attenuated protein synthesis in PANC-1 cells

**Figure 2:**
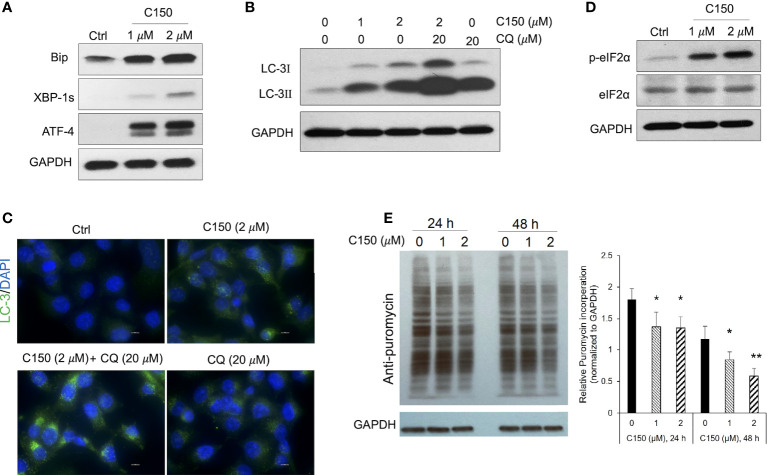
C150 induced ER stress, resulted in autophagy and attenuation of protein translation in PANC-1 cells. PANC-1 cells were treated with DMSO (Ctrl) or C150 (1 μM and 2 μM) for 24 h. **(A)** Western blots of ER stress makers. GAPDH was blotted as loading control. **(B)** Western blots of the autophagy marker LC-3. CQ, chloroquine (20 μM, 4 h treatment). **(C)** Immunofluorescence staining for LC-3 puncta. Cells were fixed and stained against LC-3 (green) and DAPI (blue). Scale bar, 10 μm. **(D)** Western blots of eIF2α and p-eIF2α. **(E)** Puromycin incorporation showing protein synthesis inhibition. PANC-1 cells were treated with 2 μM puromycin for 20 min after 24 or 48 h of treatment with C150. Total cell lysates were analyzed and blotted with anti-puromycin antibody. GAPDH was blotted as loading control. The left panel is a representative image of the Western blots. The right panel shows total band intensity normalized to GAPDH. Data are presented as mean ± SD of three independent experiments. *p < 0.05, **p < 0.01 (vs. Ctrl) by one-way ANOVA with Tukey HSD tests.

An increase in proteasome levels can be induced by ER stress (28, 29). We next investigated whether C150 treatment induced ER stress in PANC-1 cells. At 24-h treatment, C150 (1 μM and 2 μM) resulted in a profound upregulation of ER stress markers, Bip, ATF-4, and XBP-1s (**Figure 2A**). During ER stress response, autophagy is often initiated to further assist the removal of misfolded and damaged proteins (30). Our data showed that C150 treatment significantly increased LC-I and LC-3II, and the LC-3II level was further enhanced by the additional treatment of chloroquine at 20 μM for 4 h (**Figure 2B**), suggesting an increased autophagy flux by C150 treatment. The increased autophagy was further confirmed by immunostaining of LC-3 puncta in the cells (**Figure 2C**). There was a robust increase in the phosphorylation of the translation initiation factor eIF2α upon C150 treatment (**Figure 2D**). The phosphorylation of eIF2α is known to downregulate global translation, but to stimulate translation of some mRNAs such as those involved in stress responses (31). Our data in the increased expression of chaperone proteins PSMG-1 and PSMD-10 (**Figure 1H**) were consistent with this. Expecting a possible reduction of global protein synthesis (32), we then performed a puromycin incorporation assay (33) to detect protein neosynthesis (33). Puromycin can effectively incorporate into newly synthesized peptides and later be detected using Western blotting (33, 34). PANC-1 cells were treated with C150 (1 μM and 2 μM) for 24 and 48 h, and then pulse-treated with 2 μM puromycin for 20 min. Newly synthesized proteins were detected by Western blots in survived cells using an anti-puromycin antibody. Significant decreases in the levels of incorporated puromycin were detected with the treatments at either 24 or 48 h (**Figure 2E**), consistent with the eIF2α phosphorylation.

### C150 caused G2/M cell cycle arrest, induced cell senescence, and synergized with gemcitabine in PANC-1 cells

**Figure 3:**
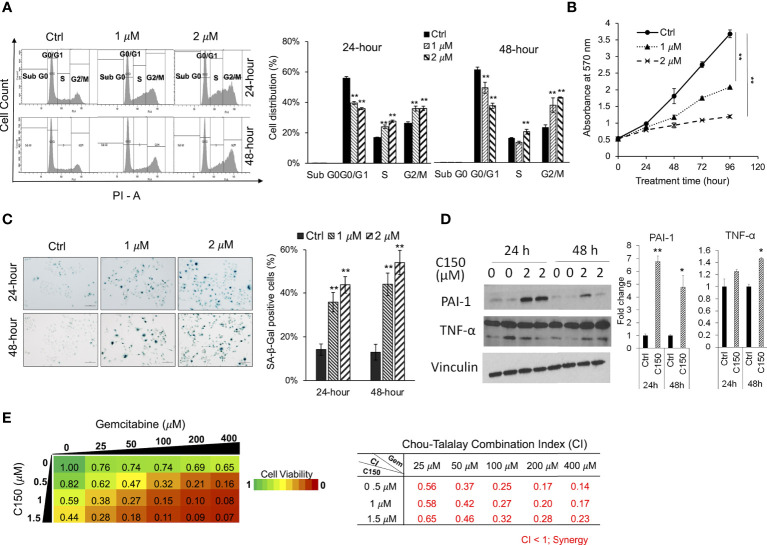
C150 caused G2/M cell cycle arrest, cellular senescence, and synergized with gemcitabine in PANC-1 cells. **(A)** Cell cycle analysis. PANC-1 cells were treated with DMSO (Ctrl) or C150 (1 μM and 2 μM) for 24 and 48 h. Cells were stained with propidium iodide (PI) and analyzed for cell cycle distributions with flow cytometry. Bar graph shows the quantification of the percentage of cells in each cell cycle. Data presented as mean ± SD of three independent experiments. **(B)** Cell growth curve. PANC-1 cells were seed at 5,000 cells per well in 96-well plates in triplicates and treated for 0, 24, 48, 72, and 96 h. Viable cells were detected by MTT assay. Data presented as mean ± SD of three experiments. **(C)** SA-β-galactosidase staining at pH 6.0 for cell senescence. Senescent cells were identified by the green-blue staining under bright field light microscopy at 200× magnification. Scale bar, 100 μm. Bar graph shows the percentage of senescent cells per imaging field with five random fields in each sample. Data presented as mean ± SD of two independent experiments each done in triplicate. **(D)** Western blots of two cell senescence markers PAI-1 and TNF-α. Band intensities were normalized to Vinculin and then compared to the untreated control. Bar graphs show fold changes versus control. Data presented as mean ± SD of two independent experiments each done in duplicate. **(E)** Heatmap of cell viabilities and combination index of C150 and gemcitabine in PANC-1 cells. PANC-1 cells were treated with C150 and gemcitabine at the indicated concentrations for 72 h. Cell viability was detected using MTT assay. Data presented as mean viability from three independent experiments each done in duplicate. The drug combination index was calculated according to the Chou-Talalay’s method. Mean CI values from three experiments were presented. *p < 0.05, **p < 0.01 (vs. Ctrl) by one-way ANOVA with Tukey HSD test.

It was reported that ER stress was able to induce cell cycle arrest (11, 35). Upon C150 treatment (1 μM and 2 μM) for 24 h and 48 h in PANC-1 cells, there was a robust increase in the cell population in the G2/M phase as demonstrated by PI cell cycle analysis (**Figure 3A**), suggesting a G2/M cell cycle arrest under C150 treatment. Cell growth curves showed a significantly reduced proliferation rate by C150 treatment (**Figure 3B**). Sustained cell cycle arrest commonly results in apoptosis and/or cell senescence (36). Our previous data have shown that C150 treatment did not induce apoptosis (22). Notably, data here showed that C150 treatments at 24 and 48 h effectively induced senescence in PANC-1 cells as indicated by the increased β-galactosidase (SA-β-galactosidase) staining at pH 6.0 (**Figure 3C**). Western blots showed that two of the known markers of cellular senescence PAI-1 and TNF-α were significantly upregulated in PANC-1 cells treated with C150 (**Figure 3D**) at 24 and 48 h, consistent with the observed senescent phenotype. Induction of senescence was reported to sensitize pancreatic cancer cells to chemotherapeutic agents (37). We found that the combination treatment of C150 with gemcitabine more effectively reduced PANC-1 cell viabilities compared to single-agent treatment (**Figure 3E**). Strong synergistic effects were shown when C150 was added to gemcitabine, with Chou-Talalay’s combination index (CI) (23) being far less than 1 (**Figure 3E**).

### C150 reduced tumor growth and increased survival in a syngeneic pancreatic cancer mouse model

**Figure 4:**
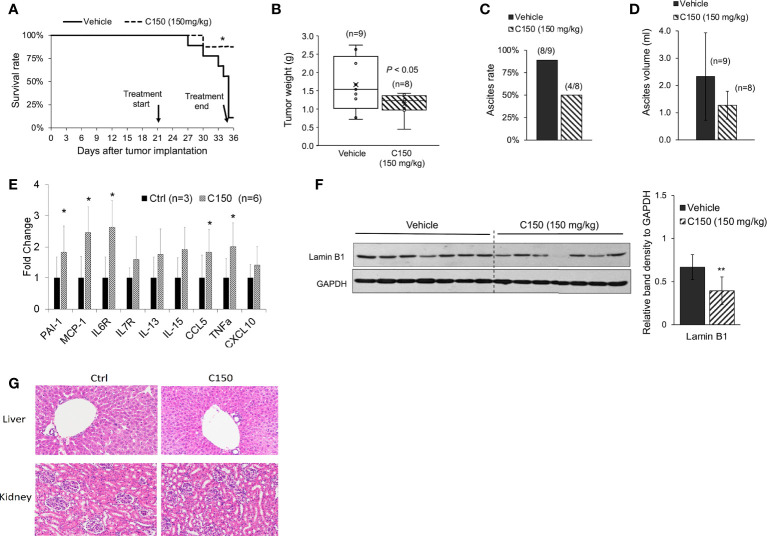
C150 treatment increased survival rate and reduced tumor growth in a syngeneic mouse model. **(A)** Kaplan–Meier survival curve of tumor-bearing mice. *p < 0.05 (vs. vehicle) by log-rank test. **(B)** Tumor weight at necropsy (*n* = 9 for vehicle, *n* = 8 for C150). *p* < 0.05 by Student’s *t*-test. **(C)** Ascites occurrence rate. **(D)** Average volume of ascites presented as mean ± SD (*n* = 9 for vehicle, *n* = 8 for C150). **(E)** RT-qPCR for mRNA expressions of senescence markers. Tumor samples from three mice in the control group and six mice in the treated group were evaluated. Results were quantified and normalized to the Ctrl group using 2^−ΔΔCt^ method with GAPDH as a housekeeping gene. **(F)** Western blotting of Lamin B1 in mouse tumor tissues. Tumors from seven individual mice in each group were analyzed. GAPDH was blotted as loading control. Bar graph shows the quantification of band density relative to GAPDH. Data presented as mean ± SD. **p < 0.01 (vs. Vehicle) by Student’s *t*-test. **(G)** H&E staining of liver and kidney tissues. Five animals from each group were examined. Representative images were shown.

A syngeneic pancreatic cancer mouse model was used to evaluate the activities of C150 *in vivo*. Compared with xenografts in immune-compromised mice, the syngeneic model preserves the intact immune functions, which plays an important role in cancer progression and responses to treatment. Pan02 mouse pancreatic cancer cells were orthotopically injected into the pancreas of C57BL/6 mice. Three weeks (21 days) after cell implantation, mice were treated with C150 (150 mg/kg) or vehicle by oral gavage three times a week for 2 weeks. Data showed that C150 treatments significantly improved the survival rate of mice at 35 days after tumor inoculation (80% survival rate in C150-treated group versus 10% in the vehicle-treated group) (**Figure 4A**). The tumor weight at necropsy was significantly reduced by C150 treatment (*n* = 8) compared to vehicle-treated controls (*n* = 9) (**Figure 4B**). Moreover, 89% (8/9) of mice in the vehicle-treated group developed ascites, whereas only 50% (4/8) in the C150 group had ascites (**Figure 4C**). In the mice that had ascites, the average volume was lower in C150-treated mice (**Figure 4D**). The expression levels of ER markers were examined in tumor tissues. Consistent with our *in vitro* data, the ER stress markers Bip, cleaved-ATF-6, ATF-4, and XBP-1s were elevated in C150-treated tumors compared to vehicle-treated controls (**Supplementary Figure 1**). A panel of cellular senescence markers were detected for their expression in the tumor samples using RT-qPCR. Data showed a significant increase in the mRNA levels of *PAI-1, MCP-1, IL6, CCL5*, and *TNF-α* in tumors treated with C150 (**Figure 4E**). Lamin B1, whose level decreases during cellular senescence (38), was also found to be decreased in C150-treated tumors (**Figure 4F**).

Because ascites developed in the tumor-bearing mice, we decided bodyweight was not a good indication of toxicity in this scenario. Instead, we examined the histology of liver and kidney at necropsy. There was no difference found in both organs between the control group and the C150-treated group (**Figure 4G**).

## Discussion

Interrupting ER homeostasis has been shown as an effective way to inhibit tumor progress because of the vital role the ER plays in cellular protein homeostasis and cell survival (39, 40). Due to high proliferation demand and hypoxic microenvironment, cancer cells are under higher endogenous ER stress, resulting in a higher endogenous activation level of UPR signaling (41). As such, pancreatic tumor tissues have higher Bip and ATF-6 expression levels than the normal pancreatic tissues (42). The high basal activation of UPR renders pancreatic cancer cells more vulnerable to the disturbance in ER homeostasis. Disrupting UPR signaling by either inhibiting or further activating it would both impede the cellular capacity to rescue ER stress, leading to catastrophic effects in the cancer cells (39, 43, 44). In agreement with this notion, our study found that C150 induced profound ER stress and further aggravated UPR signals in pancreatic cancer cells, which subsequently impeded cell proliferation, triggered cell cycle arrest, and led to pancreatic cancer cell senescence.

Findings in our study showed that C150 treatment significantly increased proteasome activity by enhancing proteasome assembly. The increased proteasome activity under ER stress is a pro-survival response of pancreatic cancer cells to restore ER proteomic homeostasis (45). However, C150-mediated increase in proteasome activity accelerated the degradation of several critical transcription factors in EMT/CSC/cell death pathways, such as Snail (22), β-catenin, Sox2, and TP53, as detected in this study. It is possible that many other important proteins in cancer cell growth/proliferation, invasion, and stemness are also influenced. The degradation of Snail and the other regulatory proteins consequently led to the inhibition of EMT and cell invasion in pancreatic cancer cells, as we previously reported (22). These results indicated that the increased proteasome activity under C150-induced ER stress may have a broad effect on degrading proteins important to pancreatic oncogenesis, resulting in comprehensive inhibition of pancreatic cancer progression through multiple pathways.

Cellular senescence is effectively evaded in pancreatic cancer due to the highly frequent loss-of-function mutations of CDKN2A and p53 (46, 47). Re-introduction of senescence has been reported as an effective approach to inhibit pancreatic cancer growth (48, 49). In our study, C150 successfully induced senescence regardless of the mutations of CDKN2A and p53 in PANC-1 cells (50). Senescence was also detected in Pan02 orthotopic mouse xenografts treated with C150, as shown by the decreased level of Lamin B1 (**Figure 4E**). Tumor growth was significantly inhibited, and survival of mice was improved. Moreover, C150-induced senescence greatly sensitized PANC-1 cells to gemcitabine treatment (**Figures 3D, E**). Therefore, C150 holds great promises in combination treatment with gemcitabine in pancreatic cancer. This synergy may be extended to other drugs, too. Further investigations are worthwhile to validate the synergistic effects in animal studies.

## Data availability statement

The original contributions presented in the study are included in the article/**Supplementary Material**. Further inquiries can be directed to the corresponding author.

## Ethics statement

The animal study was reviewed and approved by The University of Kansas Medical Center Institutional Animal Care and Use Committee.

## Author contributions

QC conceptualized and oversaw the studies and provided resources. QC and TW designed the experiments. TW performed the experiments, collected the data, interpreted the data and wrote the manuscript. PC assisted with data collection, analysis, and discussion. SW, FS, and MB participated in discussion. QC, TW, and PC have seen and can confirm the authenticity of the raw data. All authors contributed to the article and approved the submitted version.

## Funding

The present study was supported by a Lied Basic Science Pilot Project Award through the Frontiers Pilot and Collaborative Funding Program provided by the NIH/NCATS (PI: Chen), and partially by a bridging grant from the University of Kansas Research Institute (Kansas, USA), and a grant provided by the GR’s Foundation, Mosby Lincoln Foundation, Donlan Foundation (Kansas, USA), and the University of Kansas Cancer Center support grant (P30 CA168524, PI: Jensen).

## Acknowledgments

The authors would like to thank Dr. Shrikant Anant at the University of Kansas Cancer Center for providing the Pan02 cells for the animal study.

## Conflict of interest

The authors declare that the research was conducted in the absence of any commercial or financial relationships that could be construed as a potential conflict of interest.

## Publisher’s note

All claims expressed in this article are solely those of the authors and do not necessarily represent those of their affiliated organizations, or those of the publisher, the editors and the reviewers. Any product that may be evaluated in this article, or claim that may be made by its manufacturer, is not guaranteed or endorsed by the publisher.
