# Supplementary material for: Novel compound C150 inhibits pancreatic cancer through induction of ER stress and proteosome assembly
Source: Front Oncol. 2022 Oct 5;12:870473. doi: 10.3389/fonc.2022.870473 (PMC9579335; doi:10.3389/fonc.2022.870473)
Supplement: Supplementary file 1 [file Presentation_1.pdf]

Supplementary Figure 1

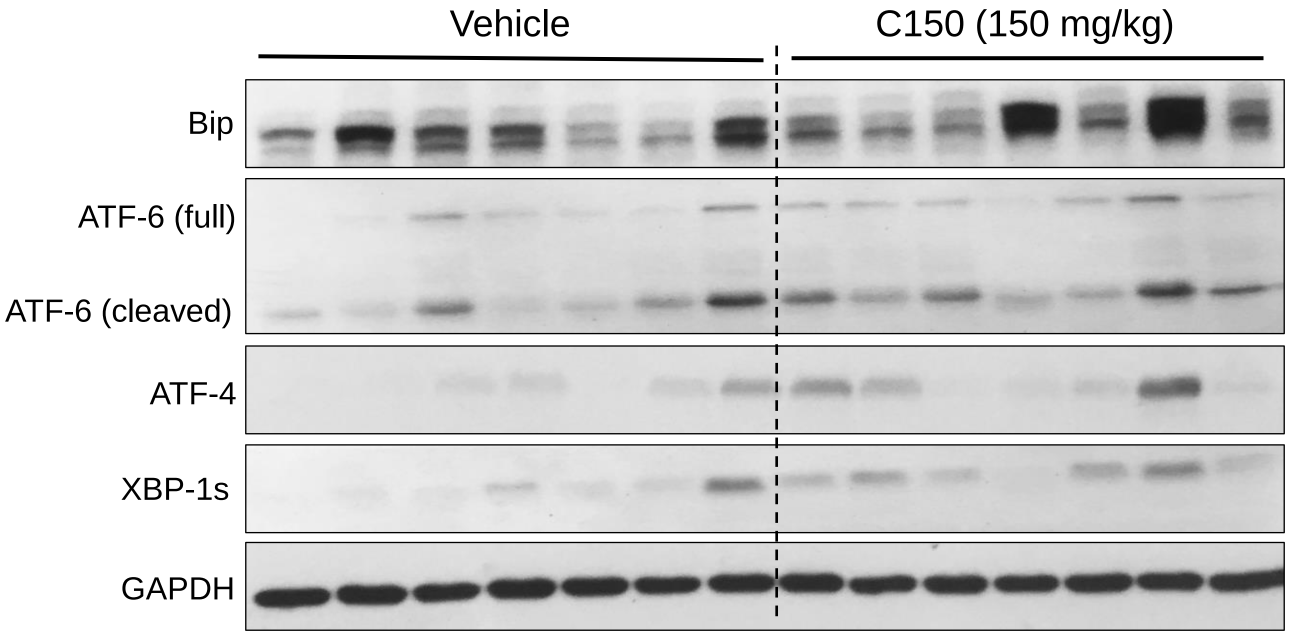

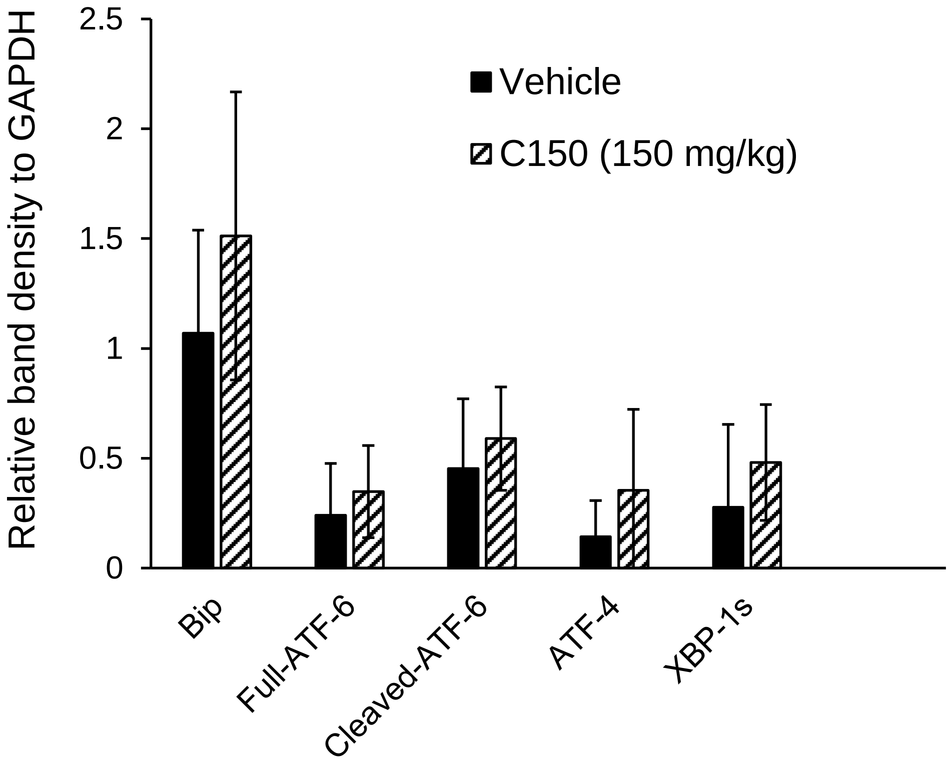

Supplementary Figure 1. Western blots of ER stress markers in Pan02 tumor tissues. Seven tumors from each group were analyzed. GAPDH is loading control. Bar graph shows the band density relative to GAPDH. Data is presented as mean ± SD.
